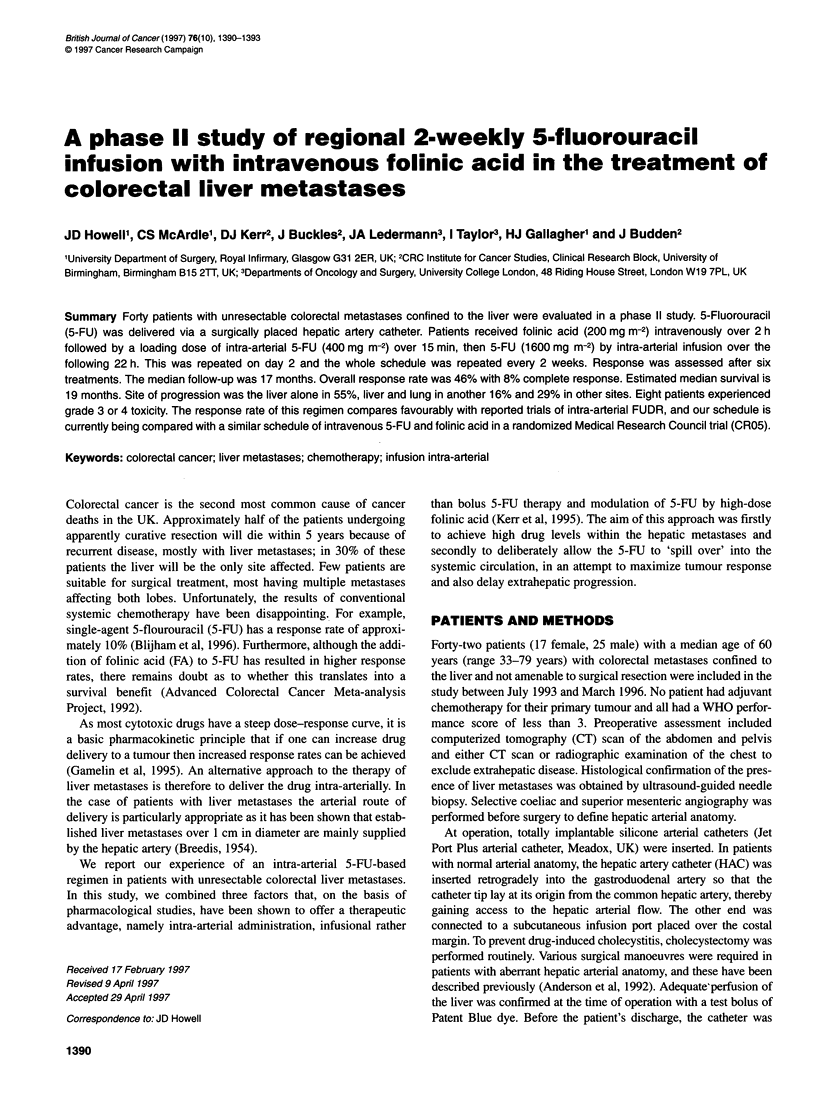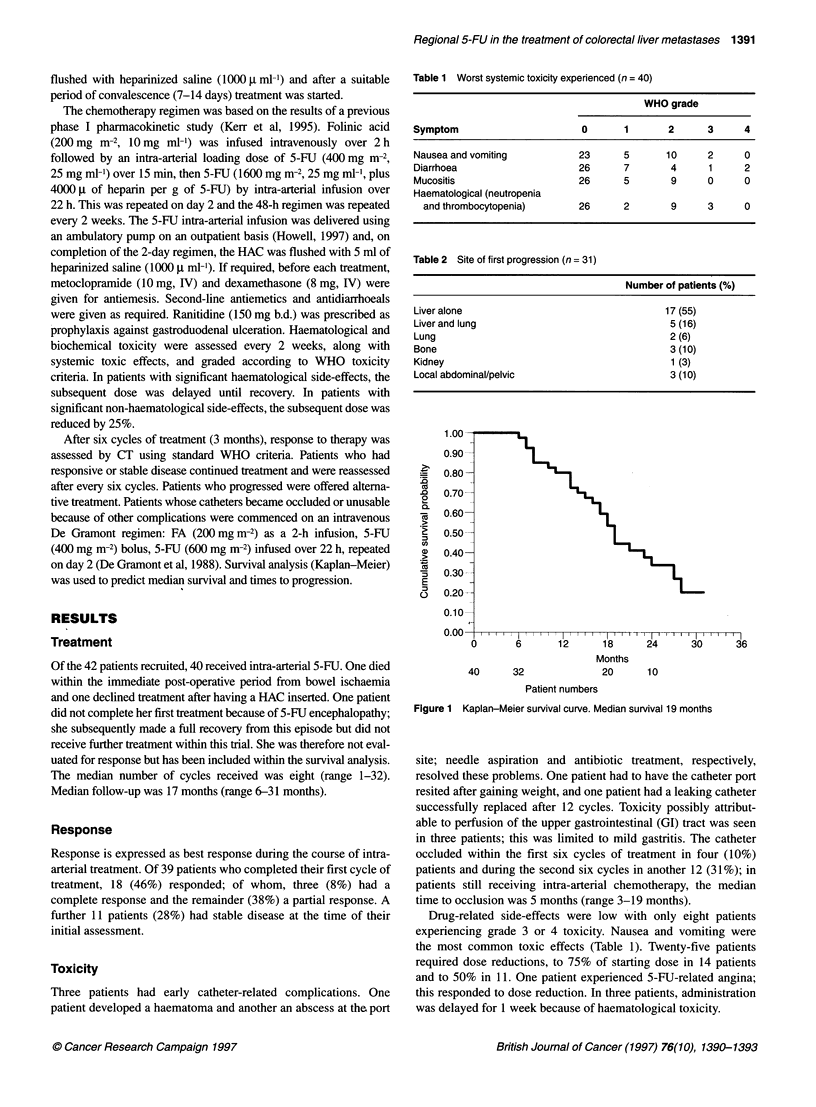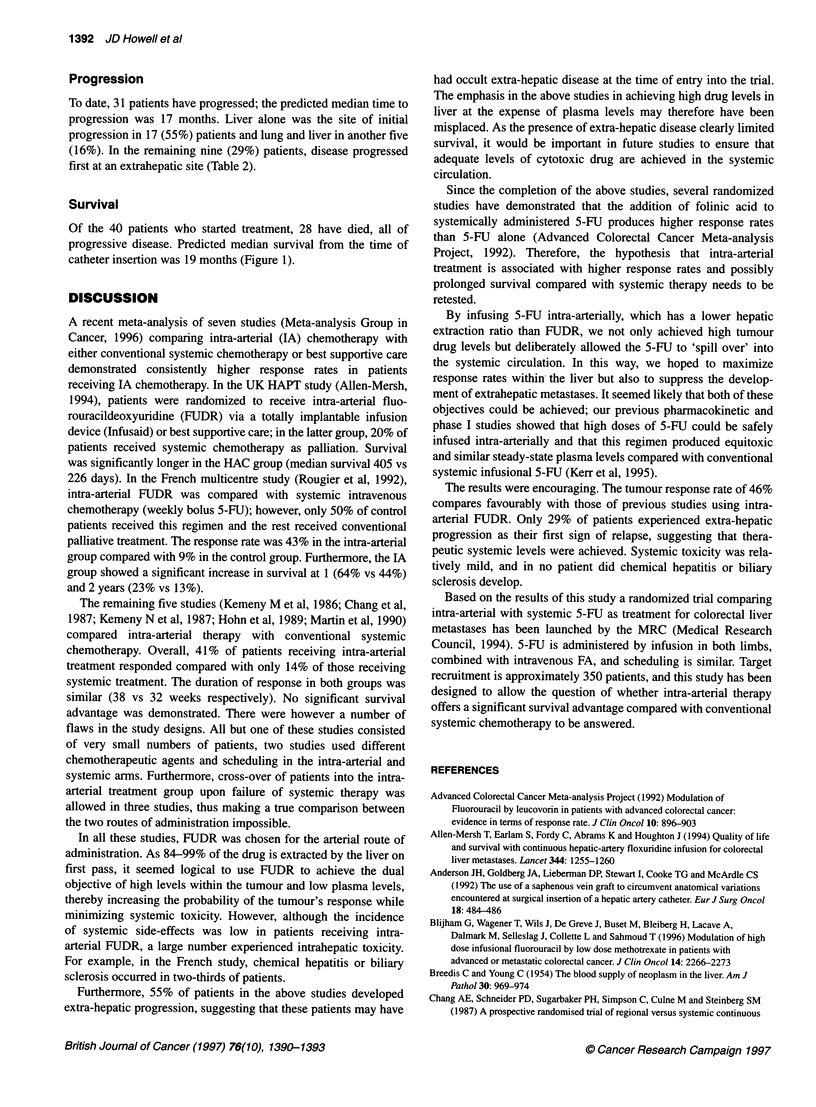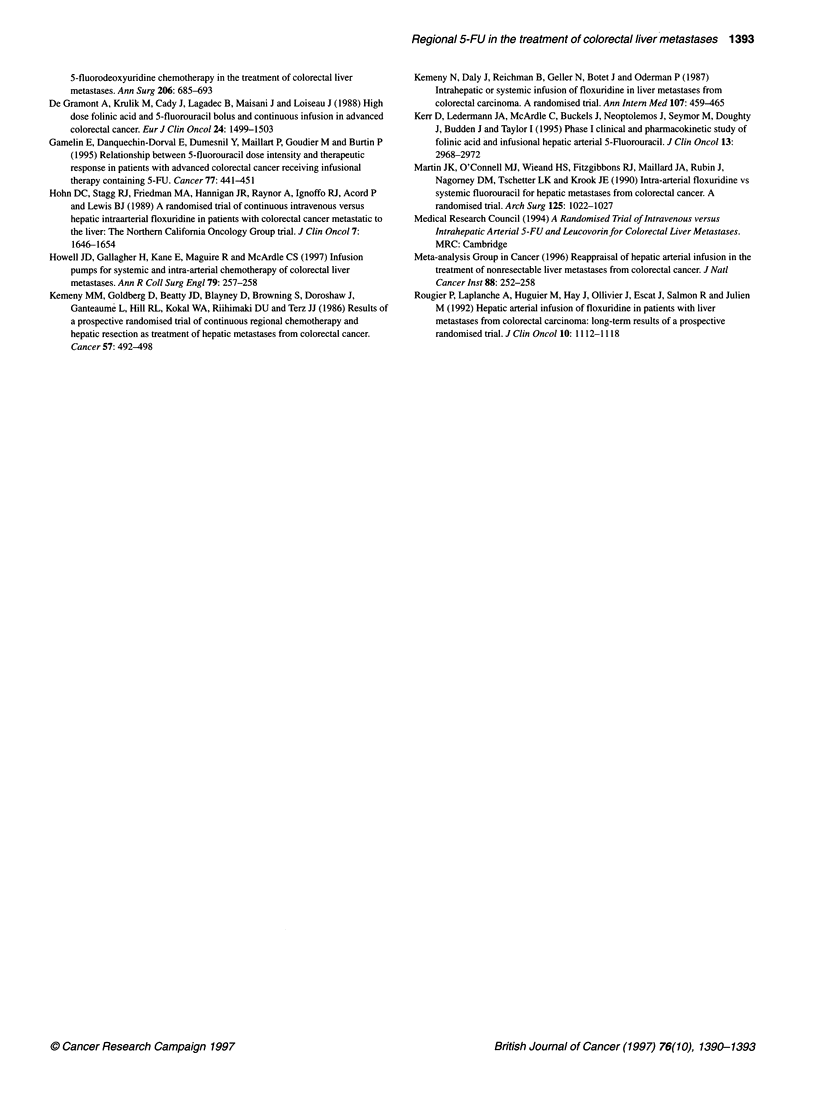# A phase II study of regional 2-weekly 5-fluorouracil infusion with intravenous folinic acid in the treatment of colorectal liver metastases.

**DOI:** 10.1038/bjc.1997.566

**Published:** 1997

**Authors:** J. D. Howell, C. S. McArdle, D. J. Kerr, J. Buckles, J. A. Ledermann, I. Taylor, H. J. Gallagher, J. Budden

**Affiliations:** University Department of Surgery, Royal Infirmary, Glasgow, UK.

## Abstract

Forty patients with unresectable colorectal metastases confined to the liver were evaluated in a phase II study. 5-Fluorouracil (5-FU) was delivered via a surgically placed hepatic artery catheter. Patients received folinic acid (200 mg m-2) intravenously over 2 h followed by a loading dose of intra-arterial 5-FU (400 mg m-2) over 15 min, then 5-FU (1600 mg m-2) by intra-arterial infusion over the following 22 h. This was repeated on day 2 and the whole schedule was repeated every 2 weeks. Response was assessed after six treatments. The median follow-up was 17 months. Overall response rate was 46% with 8% complete response. Estimated median survival is 19 months. Site of progression was the liver alone in 55%, liver and lung in another 16% and 29% in other sites. Eight patients experienced grade 3 or 4 toxicity. The response rate of this regimen compares favourably with reported trials of intra-arterial FUDR, and our schedule is currently being compared with a similar schedule of intravenous 5-FU and folinic acid in a randomized Medical Research Council trial (CR05).


British Journal of Cancer (1997) 76(10), 1390–1393
© 1997 Cancer Research Campaign

### A phase II study of regional 2-weekly 5-fluorouracil infusion with intravenous folinic acid in the treatment of colorectal liver metastases

**JD Howell[1], CS McArdle[1], DJ Kerr[2], J Buckles[2], JA Ledermann[3], I Taylor[3], HJ Gallagher[1] and J Budden[2]**

[1]University Department of Surgery, Royal Infirmary, Glasgow G31 2ER, UK; [2]CRC Institute for Cancer Studies, Clinical Research Block, University of Birmingham, Birmingham B15 2TT, UK; [3]Departments of Oncology and Surgery, University College London, 48 Riding House Street, London W19 7PL, UK

**Summary** Forty patients with unresectable colorectal metastases confined to the liver were evaluated in a phase II study. 5-Fluorouracil (5-FU) was delivered via a surgically placed hepatic artery catheter. Patients received folinic acid (200 mg $m^{-2}$) intravenously over 2 h followed by a loading dose of intra-arterial 5-FU (400 mg $m^{-2}$) over 15 min, then 5-FU (1600 mg $m^{-2}$) by intra-arterial infusion over the following 22 h. This was repeated on day 2 and the whole schedule was repeated every 2 weeks. Response was assessed after six treatments. The median follow-up was 17 months. Overall response rate was 46% with 8% complete response. Estimated median survival is 19 months. Site of progression was the liver alone in 55%, liver and lung in another 16% and 29% in other sites. Eight patients experienced grade 3 or 4 toxicity. The response rate of this regimen compares favourably with reported trials of intra-arterial FUDR, and our schedule is currently being compared with a similar schedule of intravenous 5-FU and folinic acid in a randomized Medical Research Council trial (CR05).

**Keywords:** colorectal cancer; liver metastases; chemotherapy; infusion intra-arterial

Colorectal cancer is the second most common cause of cancer deaths in the UK. Approximately half of the patients undergoing apparently curative resection will die within 5 years because of recurrent disease, mostly with liver metastases; in 30% of these patients the liver will be the only site affected. Few patients are suitable for surgical treatment, most having multiple metastases affecting both lobes. Unfortunately, the results of conventional systemic chemotherapy have been disappointing. For example, single-agent 5-flourouracil (5-FU) has a response rate of approximately 10% (Blijham et al, 1996). Furthermore, although the addition of folinic acid (FA) to 5-FU has resulted in higher response rates, there remains doubt as to whether this translates into a survival benefit (Advanced Colorectal Cancer Meta-analysis Project, 1992).

As most cytotoxic drugs have a steep dose–response curve, it is a basic pharmacokinetic principle that if one can increase drug delivery to a tumour then increased response rates can be achieved (Gamelin et al, 1995). An alternative approach to the therapy of liver metastases is therefore to deliver the drug intra-arterially. In the case of patients with liver metastases the arterial route of delivery is particularly appropriate as it has been shown that established liver metastases over 1 cm in diameter are mainly supplied by the hepatic artery (Breedis, 1954).

We report our experience of an intra-arterial 5-FU-based regimen in patients with unresectable colorectal liver metastases. In this study, we combined three factors that, on the basis of pharmacological studies, have been shown to offer a therapeutic advantage, namely intra-arterial administration, infusional rather than bolus 5-FU therapy and modulation of 5-FU by high-dose folinic acid (Kerr et al, 1995). The aim of this approach was firstly to achieve high drug levels within the hepatic metastases and secondly to deliberately allow the 5-FU to 'spill over' into the systemic circulation, in an attempt to maximize tumour response and also delay extrahepatic progression.

*Received 17 February 1997*
*Revised 9 April 1997*
*Accepted 29 April 1997*

*Correspondence to:* JD Howell

### PATIENTS AND METHODS

Forty-two patients (17 female, 25 male) with a median age of 60 years (range 33–79 years) with colorectal metastases confined to the liver and not amenable to surgical resection were included in the study between July 1993 and March 1996. No patient had adjuvant chemotherapy for their primary tumour and all had a WHO performance score of less than 3. Preoperative assessment included computerized tomography (CT) scan of the abdomen and pelvis and either CT scan or radiographic examination of the chest to exclude extrahepatic disease. Histological confirmation of the presence of liver metastases was obtained by ultrasound-guided needle biopsy. Selective coeliac and superior mesenteric angiography was performed before surgery to define hepatic arterial anatomy.

At operation, totally implantable silicone arterial catheters (Jet Port Plus arterial catheter, Meadox, UK) were inserted. In patients with normal arterial anatomy, the hepatic artery catheter (HAC) was inserted retrogradely into the gastroduodenal artery so that the catheter tip lay at its origin from the common hepatic artery, thereby gaining access to the hepatic arterial flow. The other end was connected to a subcutaneous infusion port placed over the costal margin. To prevent drug-induced cholecystitis, cholecystectomy was performed routinely. Various surgical manoeuvres were required in patients with aberrant hepatic arterial anatomy, and these have been described previously (Anderson et al, 1992). Adequate perfusion of the liver was confirmed at the time of operation with a test bolus of Patent Blue dye. Before the patient's discharge, the catheter was flushed with heparinized saline (1000 μ $ml^{-1}$) and after a suitable period of convalescence (7–14 days) treatment was started.

The chemotherapy regimen was based on the results of a previous phase I pharmacokinetic study (Kerr et al, 1995). Folinic acid (200 mg $m^{-2}$, 10 mg $ml^{-1}$) was infused intravenously over 2 h followed by an intra-arterial loading dose of 5-FU (400 mg $m^{-2}$, 25 mg $ml^{-1}$) over 15 min, then 5-FU (1600 mg $m^{-2}$, 25 mg $ml^{-1}$, plus 4000 μ of heparin per g of 5-FU) by intra-arterial infusion over 22 h. This was repeated on day 2 and the 48-h regimen was repeated every 2 weeks. The 5-FU intra-arterial infusion was delivered using an ambulatory pump on an outpatient basis (Howell, 1997) and, on completion of the 2-day regimen, the HAC was flushed with 5 ml of heparinized saline (1000 μ $ml^{-1}$). If required, before each treatment, metoclopramide (10 mg, IV) and dexamethasone (8 mg, IV) were given for antiemesis. Second-line antiemetics and antidiarrhoeals were given as required. Ranitidine (150 mg b.d.) was prescribed as prophylaxis against gastroduodenal ulceration. Haematological and biochemical toxicity were assessed every 2 weeks, along with systemic toxic effects, and graded according to WHO toxicity criteria. In patients with significant haematological side-effects, the subsequent dose was delayed until recovery. In patients with significant non-haematological side-effects, the subsequent dose was reduced by 25%.

After six cycles of treatment (3 months), response to therapy was assessed by CT using standard WHO criteria. Patients who had responsive or stable disease continued treatment and were reassessed after every six cycles. Patients who progressed were offered alternative treatment. Patients whose catheters became occluded or unusable because of other complications were commenced on an intravenous De Gramont regimen: FA (200 mg $m^{-2}$) as a 2-h infusion, 5-FU (400 mg $m^{-2}$) bolus, 5-FU (600 mg $m^{-2}$) infused over 22 h, repeated on day 2 (De Gramont et al, 1988). Survival analysis (Kaplan–Meier) was used to predict median survival and times to progression.

### RESULTS

### Treatment

Of the 42 patients recruited, 40 received intra-arterial 5-FU. One died within the immediate post-operative period from bowel ischaemia and one declined treatment after having a HAC inserted. One patient did not complete her first treatment because of 5-FU encephalopathy; she subsequently made a full recovery from this episode but did not receive further treatment within this trial. She was therefore not evaluated for response but has been included within the survival analysis. The median number of cycles received was eight (range 1–32). Median follow-up was 17 months (range 6–31 months).

### Response

Response is expressed as best response during the course of intra-arterial treatment. Of 39 patients who completed their first cycle of treatment, 18 (46%) responded; of whom, three (8%) had a complete response and the remainder (38%) a partial response. A further 11 patients (28%) had stable disease at the time of their initial assessment.

### Toxicity

Three patients had early catheter-related complications. One patient developed a haematoma and another an abscess at the port site; needle aspiration and antibiotic treatment, respectively, resolved these problems. One patient had to have the catheter port resited after gaining weight, and one patient had a leaking catheter successfully replaced after 12 cycles. Toxicity possibly attributable to perfusion of the upper gastrointestinal (GI) tract was seen in three patients; this was limited to mild gastritis. The catheter occluded within the first six cycles of treatment in four (10%) patients and during the second six cycles in another 12 (31%); in patients still receiving intra-arterial chemotherapy, the median time to occlusion was 5 months (range 3–19 months).

Drug-related side-effects were low with only eight patients experiencing grade 3 or 4 toxicity. Nausea and vomiting were the most common toxic effects (Table 1). Twenty-five patients required dose reductions, to 75% of starting dose in 14 patients and to 50% in 11. One patient experienced 5-FU-related angina; this responded to dose reduction. In three patients, administration was delayed for 1 week because of haematological toxicity.

**Table 1** Worst systemic toxicity experienced (*n* = 40)

| | WHO grade | | | | |
|---|---|---|---|---|---|
| **Symptom** | **0** | **1** | **2** | **3** | **4** |
| Nausea and vomiting | 23 | 5 | 10 | 2 | 0 |
| Diarrhoea | 26 | 7 | 4 | 1 | 2 |
| Mucositis | 26 | 5 | 9 | 0 | 0 |
| Haematological (neutropenia and thrombocytopenia) | 26 | 2 | 9 | 3 | 0 |

**Table 2** Site of first progression (*n* = 31)

| | Number of patients (%) |
|---|---|
| Liver alone | 17 (55) |
| Liver and lung | 5 (16) |
| Lung | 2 (6) |
| Bone | 3 (10) |
| Kidney | 1 (3) |
| Local abdominal/pelvic | 3 (10) |

**Figure 1** Kaplan–Meier survival curve. Median survival 19 months

### Progression

To date, 31 patients have progressed; the predicted median time to progression was 17 months. Liver alone was the site of initial progression in 17 (55%) patients and lung and liver in another five (16%). In the remaining nine (29%) patients, disease progressed first at an extrahepatic site (Table 2).

### Survival

Of the 40 patients who started treatment, 28 have died, all of progressive disease. Predicted median survival from the time of catheter insertion was 19 months (Figure 1).

### DISCUSSION

A recent meta-analysis of seven studies (Meta-analysis Group in Cancer, 1996) comparing intra-arterial (IA) chemotherapy with either conventional systemic chemotherapy or best supportive care demonstrated consistently higher response rates in patients receiving IA chemotherapy. In the UK HAPT study (Allen-Mersh, 1994), patients were randomized to receive intra-arterial fluorouracildeoxyuridine (FUDR) via a totally implantable infusion device (Infusaid) or best supportive care; in the latter group, 20% of patients received systemic chemotherapy as palliation. Survival was significantly longer in the HAC group (median survival 405 vs 226 days). In the French multicentre study (Rougier et al, 1992), intra-arterial FUDR was compared with systemic intravenous chemotherapy (weekly bolus 5-FU); however, only 50% of control patients received this regimen and the rest received conventional palliative treatment. The response rate was 43% in the intra-arterial group compared with 9% in the control group. Furthermore, the IA group showed a significant increase in survival at 1 (64% vs 44%) and 2 years (23% vs 13%).

The remaining five studies (Kemeny M et al, 1986; Chang et al, 1987; Kemeny N et al, 1987; Hohn et al, 1989; Martin et al, 1990) compared intra-arterial therapy with conventional systemic chemotherapy. Overall, 41% of patients receiving intra-arterial treatment responded compared with only 14% of those receiving systemic treatment. The duration of response in both groups was similar (38 vs 32 weeks respectively). No significant survival advantage was demonstrated. There were however a number of flaws in the study designs. All but one of these studies consisted of very small numbers of patients, two studies used different chemotherapeutic agents and scheduling in the intra-arterial and systemic arms. Furthermore, cross-over of patients into the intra-arterial treatment group upon failure of systemic therapy was allowed in three studies, thus making a true comparison between the two routes of administration impossible.

In all these studies, FUDR was chosen for the arterial route of administration. As 84–99% of the drug is extracted by the liver on first pass, it seemed logical to use FUDR to achieve the dual objective of high levels within the tumour and low plasma levels, thereby increasing the probability of the tumour's response while minimizing systemic toxicity. However, although the incidence of systemic side-effects was low in patients receiving intra-arterial FUDR, a large number experienced intrahepatic toxicity. For example, in the French study, chemical hepatitis or biliary sclerosis occurred in two-thirds of patients.

Furthermore, 55% of patients in the above studies developed extra-hepatic progression, suggesting that these patients may have had occult extra-hepatic disease at the time of entry into the trial. The emphasis in the above studies in achieving high drug levels in liver at the expense of plasma levels may therefore have been misplaced. As the presence of extra-hepatic disease clearly limited survival, it would be important in future studies to ensure that adequate levels of cytotoxic drug are achieved in the systemic circulation.

Since the completion of the above studies, several randomized studies have demonstrated that the addition of folinic acid to systemically administered 5-FU produces higher response rates than 5-FU alone (Advanced Colorectal Cancer Meta-analysis Project, 1992). Therefore, the hypothesis that intra-arterial treatment is associated with higher response rates and possibly prolonged survival compared with systemic therapy needs to be retested.

By infusing 5-FU intra-arterially, which has a lower hepatic extraction ratio than FUDR, we not only achieved high tumour drug levels but deliberately allowed the 5-FU to 'spill over' into the systemic circulation. In this way, we hoped to maximize response rates within the liver but also to suppress the development of extrahepatic metastases. It seemed likely that both of these objectives could be achieved; our previous pharmacokinetic and phase I studies showed that high doses of 5-FU could be safely infused intra-arterially and that this regimen produced equitoxic and similar steady-state plasma levels compared with conventional systemic infusional 5-FU (Kerr et al, 1995).

The results were encouraging. The tumour response rate of 46% compares favourably with those of previous studies using intra-arterial FUDR. Only 29% of patients experienced extra-hepatic progression as their first sign of relapse, suggesting that therapeutic systemic levels were achieved. Systemic toxicity was relatively mild, and in no patient did chemical hepatitis or biliary sclerosis develop.

Based on the results of this study a randomized trial comparing intra-arterial with systemic 5-FU as treatment for colorectal liver metastases has been launched by the MRC (Medical Research Council, 1994). 5-FU is administered by infusion in both limbs, combined with intravenous FA, and scheduling is similar. Target recruitment is approximately 350 patients, and this study has been designed to allow the question of whether intra-arterial therapy offers a significant survival advantage compared with conventional systemic chemotherapy to be answered.

### REFERENCES

Advanced Colorectal Cancer Meta-analysis Project (1992) Modulation of Fluorouracil by leucovorin in patients with advanced colorectal cancer: evidence in terms of response rate. *J Clin Oncol* **10**: 896–903

Allen-Mersh T, Earlam S, Fordy C, Abrams K and Houghton J (1994) Quality of life and survival with continuous hepatic-artery floxuridine infusion for colorectal liver metastases. *Lancet* **344**: 1255–1260

Anderson JH, Goldberg JA, Lieberman DP, Stewart I, Cooke TG and McArdle CS (1992) The use of a saphenous vein graft to circumvent anatomical variations encountered at surgical insertion of a hepatic artery catheter. *Eur J Surg Oncol* **18**: 484–486

Blijham G, Wagener T, Wils J, De Greve J, Buset M, Bleiberg H, Lacave A, Dalmark M, Selleslag J, Collette L and Sahmoud T (1996) Modulation of high dose infusional fluorouracil by low dose methotrexate in patients with advanced or metastatic colorectal cancer. *J Clin Oncol* **14**: 2266–2273

Breedis C and Young C (1954) The blood supply of neoplasm in the liver. *Am J Pathol* **30**: 969–974

Chang AE, Schneider PD, Sugarbaker PH, Simpson C, Culne M and Steinberg SM (1987) A prospective randomised trial of regional versus systemic continuous

© Cancer Research Campaign 1997

5-fluorodeoxyuridine chemotherapy in the treatment of colorectal liver metastases. *Ann Surg* **206**: 685–693

De Gramont A, Krulik M, Cady J, Lagadec B, Maisani J and Loiseau J (1988) High dose folinic acid and 5-fluorouracil bolus and continuous infusion in advanced colorectal cancer. *Eur J Clin Oncol* **24**: 1499–1503

Gamelin E, Danquechin-Dorval E, Dumesnil Y, Maillart P, Goudier M and Burtin P (1995) Relationship between 5-fluorouracil dose intensity and therapeutic response in patients with advanced colorectal cancer receiving infusional therapy containing 5-FU. *Cancer* **77**: 441–451

Hohn DC, Stagg RJ, Friedman MA, Hannigan JR, Raynor A, Ignoffo RJ, Acord P and Lewis BJ (1989) A randomised trial of continuous intravenous versus hepatic intraarterial floxuridine in patients with colorectal cancer metastatic to the liver: The Northern California Oncology Group trial. *J Clin Oncol* **7**: 1646–1654

Howell JD, Gallagher H, Kane E, Maguire R and McArdle CS (1997) Infusion pumps for systemic and intra-arterial chemotherapy of colorectal liver metastases. *Ann R Coll Surg Engl* **79**: 257–258

Kemeny MM, Goldberg D, Beatty JD, Blayney D, Browning S, Doroshaw J, Ganteaumė L, Hill RL, Kokal WA, Riihimaki DU and Terz JJ (1986) Results of a prospective randomised trial of continuous regional chemotherapy and hepatic resection as treatment of hepatic metastases from colorectal cancer. *Cancer* **57**: 492–498

Kemeny N, Daly J, Reichman B, Geller N, Botet J and Oderman P (1987) Intrahepatic or systemic infusion of floxuridine in liver metastases from colorectal carcinoma. A randomised trial. *Ann Intern Med* **107**: 459–465

Kerr D, Ledermann JA, McArdle C, Buckels J, Neoptolemos J, Seymor M, Doughty J, Budden J and Taylor I (1995) Phase I clinical and pharmacokinetic study of folinic acid and infusional hepatic arterial 5-Fluorouracil. *J Clin Oncol* **13**: 2968–2972

Martin JK, O'Connell MJ, Wieand HS, Fitzgibbons RJ, Maillard JA, Rubin J, Nagorney DM, Tschetter LK and Krook JE (1990) Intra-arterial floxuridine vs systemic fluorouracil for hepatic metastases from colorectal cancer. A randomised trial. *Arch Surg* **125**: 1022–1027

Medical Research Council (1994) *A Randomised Trial of Intravenous versus Intrahepatic Arterial 5-FU and Leucovorin for Colorectal Liver Metastases*. MRC: Cambridge

Meta-analysis Group in Cancer (1996) Reappraisal of hepatic arterial infusion in the treatment of nonresectable liver metastases from colorectal cancer. *J Natl Cancer Inst* **88**: 252–258

Rougier P, Laplanche A, Huguier M, Hay J, Ollivier J, Escat J, Salmon R and Julien M (1992) Hepatic arterial infusion of floxuridine in patients with liver metastases from colorectal carcinoma: long-term results of a prospective randomised trial. *J Clin Oncol* **10**: 1112–1118

© Cancer Research Campaign 1997